# Castleman disease versus lymphoma in neck lymph nodes: a comparative study using contrast-enhanced CT

**DOI:** 10.1186/s40644-018-0163-7

**Published:** 2018-08-16

**Authors:** Jie Li, Jia Wang, Zhitao Yang, Hexiang Wang, Junyi Che, Wenjian Xu

**Affiliations:** 1 Department of Radiology, The Affiliated Hospital of Qingdao University, 16 Jiangsu Road, Qingdao, Shandong China; 2 Department of Ultrasound, Qingdao Women and Children Hospital, Qingdao, Shandong China; 3 Department of Radiology, Qingdao Municipal Hospital, Qingdao, Shandong China

**Keywords:** Neck, Castleman disease, Lymphoma, Contrast-enhanced CT

## Abstract

**Background:**

The purpose of this study was to determine the contrast-enhanced CT characteristics for differentiating between Castleman disease (CD) and lymphoma in neck lymph nodes.

**Methods:**

This retrospective study evaluated the number (solitary or multiple), strength of contrast-enhancement, type of contrast-enhancement, surrounding vessels, contrast-enhanced Hounsfield unit (HU) values, and anatomical distributions of lymph nodes in 34 patients with confirmed CD and 55 patients with newly diagnosed untreated lymphoma. Independent *t*-tests, receiver operating characteristic (ROC) curve analysis, and chi-square tests were used to evaluate the variables and CT features.

**Results:**

Several significant differences were found between CD and lymphoma. The interval between first contrast-enhanced CT and biopsy/surgery was significantly longer in the CD group (mean 72 ± 105 days, median 60 days) than in the lymphoma patients (mean 30 ± 2 days, median 12 days; *p* = 0.015). The lymphoma patients presented significantly more often with fatigue and fever (*p* = 0.023 and *p* = 0.016 respectively) than did the CD subjects. HU values of nodules after enhancement were significantly higher in the CD patients than in the lymphoma patients. In cases involving multiple lymph nodes, in all the CD cases, all affected nodes were located in only the left or right side of the neck, not bilaterally. ROC analysis showed a significant difference in contrast-enhanced CT attenuation values between lymphoma and CD (*p* < 0.001, area under the curve = 0.954), with a cut-off value of 92.5 HU. We constructed a decision tree according to these imaging characteristics.

**Conclusions:**

Contrast-enhanced CT can be useful for differentiating between CD and lymphoma.

## Background

The incidence of Castleman disease (CD) has increased in recent years, with this phenomenon having been attributed to factors such as an increase in acquired immunodeficiency syndrome (AIDS), serum interleukin-6 (IL-6) levels, and/or human herpes virus-8 (HHV-8) [1–3].

CD can mimic other pathology, and especially when it is located in the neck, it often shares similar clinical presentations and imaging characteristics to lymphoma, such as a progressive growing lesion and enlarged lymph nodes on imaging [4, 5]. In addition, because of the low incidence of CD, it can be easy for the radiologist to misdiagnose it as lymphoma, without raising suspicion of CD. Although the two conditions share some similar clinical presentations and imaging characteristics, the treatments for lymphoma and CD are quite different [6, 7]. Surgical resection is suitable for patients with non-multicentric type CD [6, 8–10], while the treatment of choice for lymphoma is chemotherapy and radiotherapy [7].

To achieve an accurate diagnosis and appropriate treatment, it would be helpful if these two conditions could be accurately differentiated on imaging. To our knowledge, very few studies have assessed the diagnostic performance of contrast-enhanced CT for differentiating CD from lymphoma [11–13]. Therefore, the purpose of this study was to compare the characteristics of CD and lymphoma on contrast-enhanced CT, to promote appropriate treatment.

## Methods

### Patients

This retrospective study was approved by our institutional review boards and the requirement for informed consent was waived. The pathology database of our institution was searched for cases of CD and cases of lymphoma occurring between December 2012 and May 2018. A total of 44 patients with CD in the neck were retrieved, although 4 cases were excluded because of the absence of a contrast-enhanced CT exam, and 6 cases were excluded because of neoplastic disease. A total of 113 patients with lymphoma were retrieved, but 58 cases were excluded from the study because of the lack of a contrast-enhanced CT exam, previous treatments, or other neoplasms occurring in other parts of the body. A final total of 89 patients were therefore included in this retrospective study, with 34 patients with CD forming a CD group, and 55 patients with newly diagnosed lymphomas involving neck lymph nodes forming a lymphoma group. All of the 89 patients were confirmed by pathological examination.

The 34 patients with CD showed no evidence of neoplastic disease or infection. All of the 34 CD patients underwent a contrast-enhanced CT exam, and 30 patients also received an ultrasound examination.

The 55 patients with newly diagnosed and untreated lymphoma all underwent a contrast-enhanced CT exam, and 46 patients also received an ultrasound examination.

### Imaging technique

CT imaging was performed using a 64-row multidetector CT scanner (Sensation 64; Siemens, Erlangen, Germany) or a 128-row CT scanner (Discovery 750 HDCT; GE Healthcare, Milwaukee, WI, USA). All of the patients were scanned using the same parameters: 3-mm slice-thickness reconstruction, 23–25-cm field of view, 120-kV voltage, 200–300-mA current, and 256 × 256 matrix. An iodinated contrast agent (Ultravist 370; Bayer HealthCare LLC, Leverkusen, Germany; 2.0 ml/kg) was administered via the antecubital vein by an automated injector system (CT 9000; LiebeleFlarsheim, Cincinnati, OH, USA) at a rate of 2.5 ml/s. The contrast-enhanced imaging phase was started 30 s after injection of the iodinated contrast agent.

### Image analysis

Two head and neck radiologists with 15 years and 11 years of experience blinded to the final diagnosis independently reviewed all CT images on a picture archiving and communication system (PACS), and recorded the number of nodules (solitary or multiple), and their size, location, degree of enhancement, and average contrast-enhanced Hounsfield unit (HU) value. On the contrast-enhanced CT imaging, the degree of enhancement was categorized as mild, moderate, or marked enhancement, with the standard used for assessment of enhancement being: mild enhancement, the enhancement of the nodule was close to that of adjacent muscles; moderate enhancement, enhancement slightly higher than the adjacent muscles; marked enhancement, enhancement markedly higher than in the muscles. The average HU value was measured from a small region of interest (ROI) placed in the middle of a node and areas of necrosis were avoided. Any discrepancies in interpretation between the observers were resolved by consensus.

### Statistical analysis

The anatomical distributions and enhancement features of the involved lymph nodes of the two patient groups were analyzed statistically using the SPSS statistical package (version 13.0; SPSS Inc., Chicago, IL, USA). Age at the time of diagnosis and interval from the first contrast-enhanced CT to stereobiopsy or surgery were expressed as the mean ± standard deviation and compared using independent *t*-tests. Receiver operating characteristic (ROC) curve analysis was used to assess the diagnostic value of the CT attenuation values, including determination of an appropriate cut-off value. Differences in the categorical variables between the CD and lymphoma groups were analyzed using chi-square tests. Sensitivity, specificity, positive predictive value (PPV), negative predictive value (NPV), and Youden Index were also analyzed. The Youden index was calculated as (sensitivity + specificity − 1). A *p* value of less than 0.05 was considered statistically significant.

## Results

### Patient and clinical data

The patient recruitment data are summarized in Table 1. The interval between the first contrast-enhanced CT and biopsy/surgery was significantly longer in the CD group (mean 72 ± 105 days, median 60 days) than in the lymphoma patients (mean 30 ± 2 days, median 12 days; *p* = 0.015), while there was no statistically significant difference in sex and age between the two groups.

**Table 1:** Patient recruitment data

	CD	lymphoma
NO. of patients	34	55
Sex	12 female,	33 female,
	22 male	22 male
Age	38 ± 9 years (median 35 years)	43 ± 8 years (median 39 years)
Interval from the first contrast-enhanced CT to stereobiopsy or surgery	72 ± 105 days (median 60 days)	30 ± 2 days (median 12 days)
	min. 5 day	min. 1 day
	max. 250 days	max. 30 days

Table 2 lists the various clinical manifestations presented by the CD and lymphoma patients. The most common manifestation in the two subject groups was a progressive growing mass or masses in the neck. Lymphoma patients presented with fatigue and fever significantly more often (*p* = 0.023, *p =* 0.016 respectively) than CD subjects. CD patients presented with neck pain significantly more often than lymphoma patients (*p* = 0.009).

**Table 2:** Clinical presentation

Clinical symptoms	CD (NO. of patients)	lymphoma (NO. of patients)
progressive growing mass or masses	29	40
fatigue	2	45
fever	6	35
neck pain	24	6
anemia	0	15

NO. number

### Contrast-enhanced CT findings

**Fig. 1:**
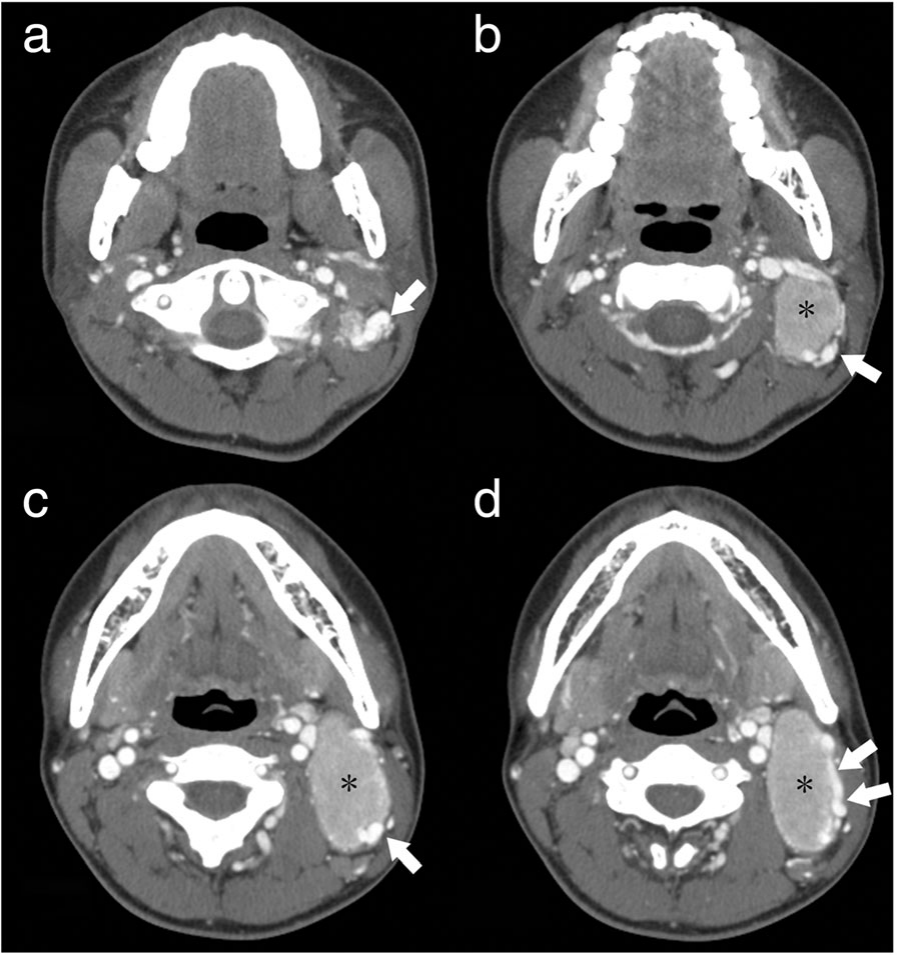
A 29-year-old man who presented with hyaline vascular type Castleman disease. **a**-**d** Contrast-enhanced CT images of the neck show a solitary nodule (black *) with a marked enhancement pattern and dilated and tortuous vessels in the periphery of the nodule (white arrows)

**Fig. 2:**
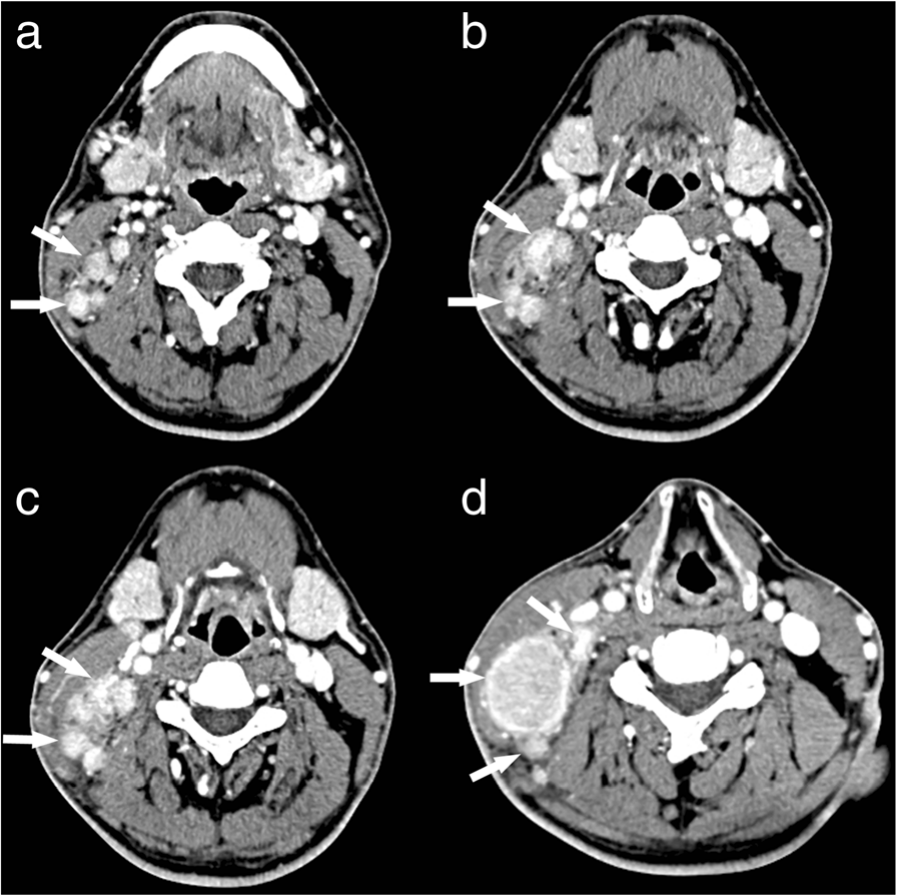
A 37-year-old man who presented with hyaline vascular type Castleman disease. **a**-**d** Contrast-enhanced CT images of the neck show multiple nodules with a marked enhancement pattern (white arrows), with all of the nodules located on the right side of the neck. There are no enlarged lymph nodes on the left side of the neck

**Fig. 3:**
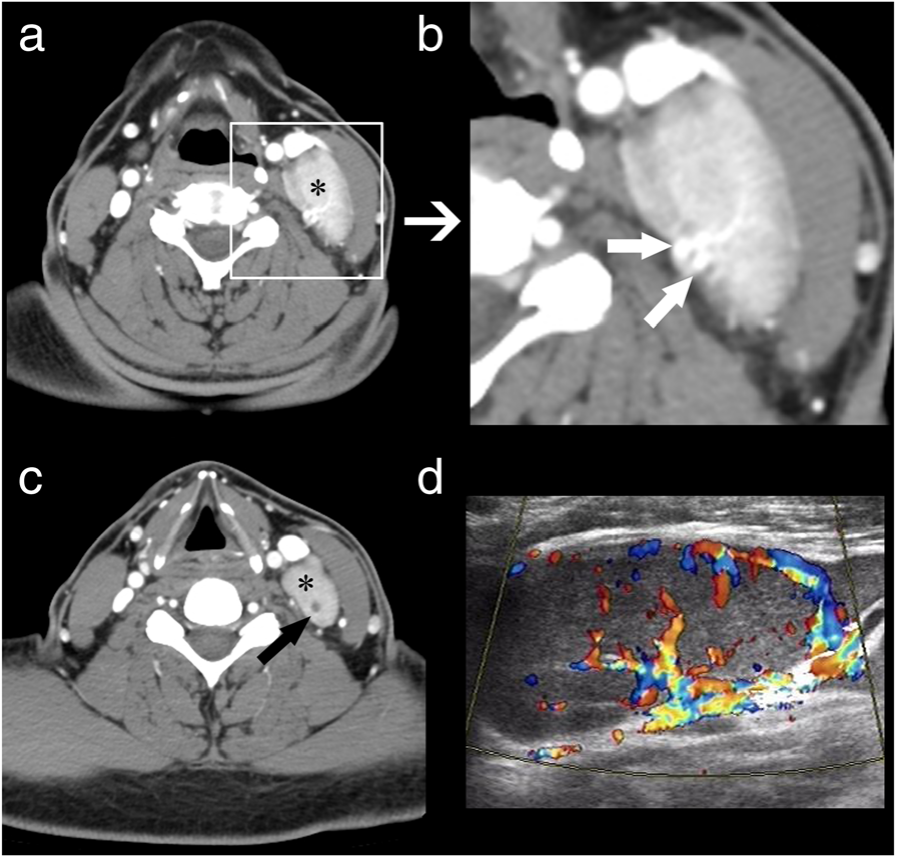
A 44-year-old man who presented with hyaline vascular type Castleman disease. **a**, **c** Contrast-enhanced CT images of the neck show multiple nodules (black *) with marked enhancement patterns. **b** Dilated and tortuous vessels are observable around the nodules. **c** An intra-nodular cystic region is also observable (black arrow). **d** Spectral Doppler ultrasound imaging shows plentiful blood flow

The diagnostic contrast-enhanced CT characteristics of both groups are summarized in Table 3. At the time of the initial contrast-enhanced CT, 82.4% of CD patients presented with a solitary nodule (Fig. 1a–d). CD typically appeared as marked enhancement in 85.3% of cases (Fig. 2a–d), with moderate enhancement being observed in 14.7% of cases. CD presented as multiple nodules in 6 (17.6%) cases, with all of the multiple nodules being located in only the left or right side of the neck (Fig. 2a–d), not bilaterally. Homogeneous enhancement was present in 88.2% of cases, with only one case of CD showing the presence of necrosis (Fig. 3c).

**Table 3:** Contrast-enhanced CT findings in CD and lymphoma at the time of initial evaluation

Characteristics	CD	lymphoma	*P* value
Type of lesions, No. (%)			< 0.001
solitary nodule	28(82.4%)	12(21.8%)	
multiple nodules	6(17.6%)	43(78.2%)	
Vessel(s) entering nodule(s)	23(67.6%)	0(0.0%)	< 0.001
Dilated and tortuous vessels in the periphery of nodule(s)	12(35.3%)	1(1.8%)	< 0.001
Type of enhancement, No. (%)			0.684
homogeneous	30(88.2%)	50(90.9%)	
nonhomogeneous	4(11.8%)	5(9.1%)	
presence of necrosis	1(2.9%)	2(3.6%)	
Degree of enhancement, No. (%)			< 0.001
mild enhancement	0(0.0%)	12(21.8%)	
moderate enhancement	5(14.7%)	43(78.2%)	
marked enhancement	29(85.3%)	0(0.0%)	
Hounsfield units (HU)	108.6 ± 20.5	75.6 ± 13.2	0.033
Margin, No. (%)			0.233
well-defined margin	28(82.1%)	50(90.9%)	
ill-defined margin	6(17.6%)	5(9.1%)	
Calcification	1(2.9%)	1(1.8%)	0.621

**Fig. 4:**
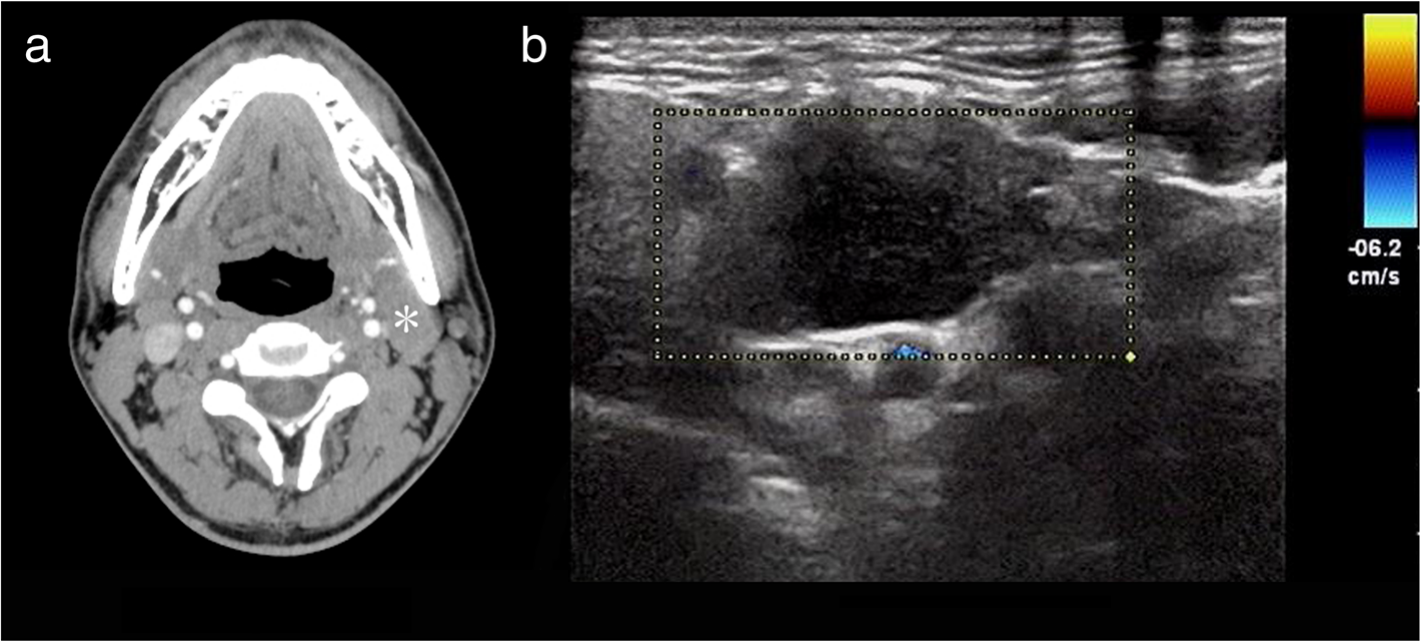
A 47-year-old woman who presented with non-Hodgkin’s lymphoma. **a** A contrast-enhanced CT image of the neck shows a solitary nodule (white *) with a mild enhancement pattern. **b** Spectral Doppler ultrasound imaging shows no blood flow

**Fig. 5:**
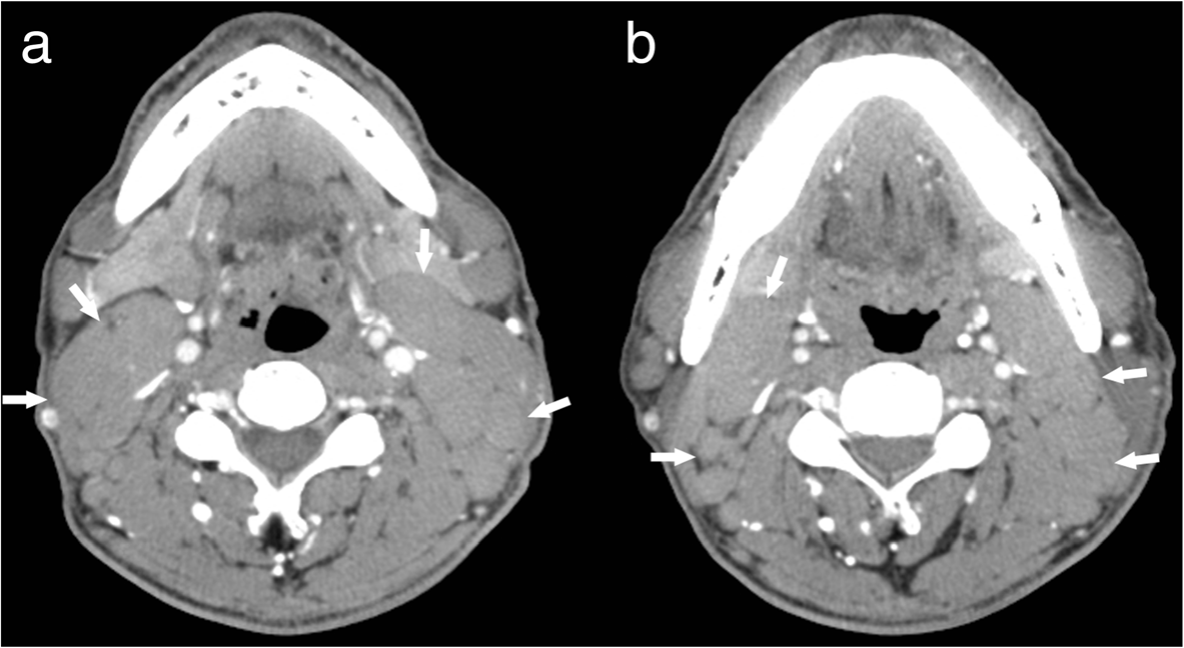
A 35-year-old man who presented with non-Hodgkin’s lymphoma. **a**-**b** Contrast-enhanced CT images of the neck show multiple nodules with mild enhancement patterns (white arrows), with the nodules being located on both sides of the neck (white arrows)

**Fig. 6:**
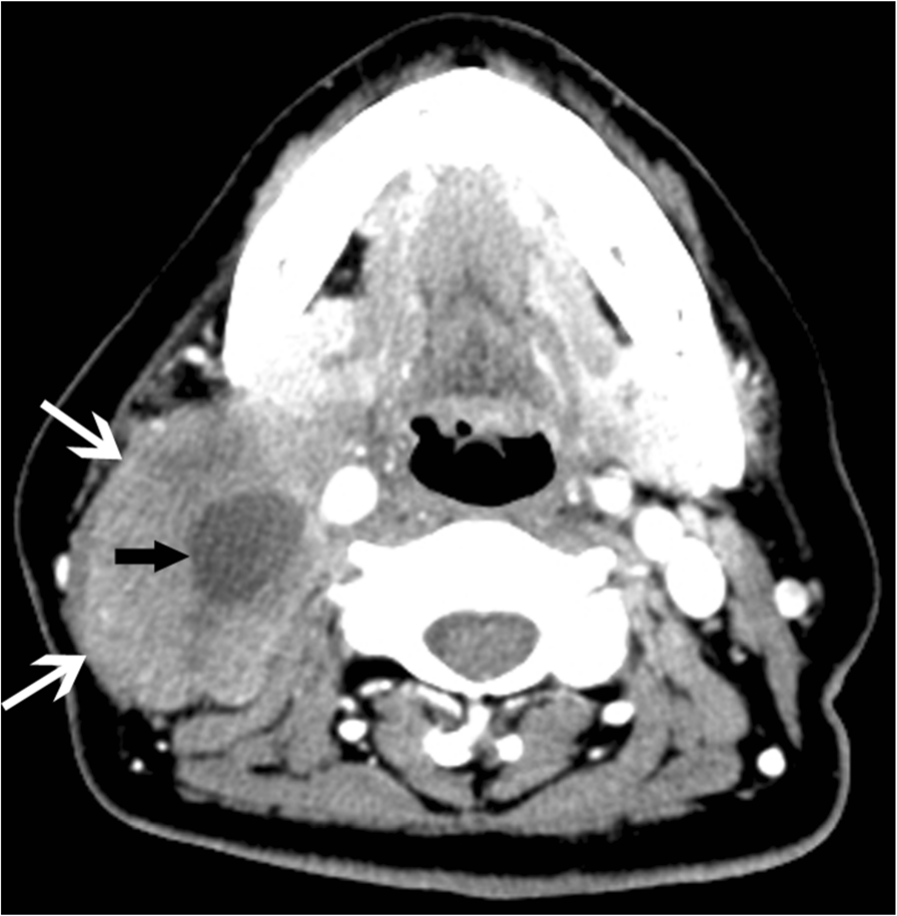
A 47-year-old woman who presented with non-Hodgkin’s lymphoma. A contrast-enhanced CT image of the neck shows an ill-defined margin (white arrow) and a region of liquefaction and necrosis with a mild enhancement pattern in the interior of the mass (black arrow)

In the lymphoma subjects, multiple nodules were present in 78.2% of cases and solitary nodules in 21.8%. Moderate enhancement was detected in 43 cases (46.3%) and mild enhancement in 21.8% (Fig. 4a, b). In 38 of 43 lymphoma cases presenting with multiple nodules, the multiple nodules were located bilaterally in the sides of the neck (Fig. 5a, b). The lymphomas mostly showed homogeneous enhancement (90.9%), with non-homogeneous enhancement being detected in only 9.1% of cases. The presence of necrosis was rare (3.6%; Fig. 6). Ill-defined margins were more often detected in CD (17.6%) than in lymphoma (9.1%; Fig. 2a–d).

**Fig. 7:**
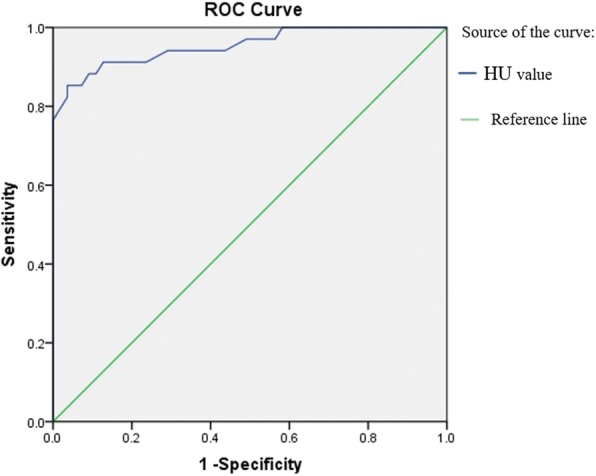
Receiver operating characteristic curves for attenuation values in differentiating CD from lymphoma

**Fig. 8:**
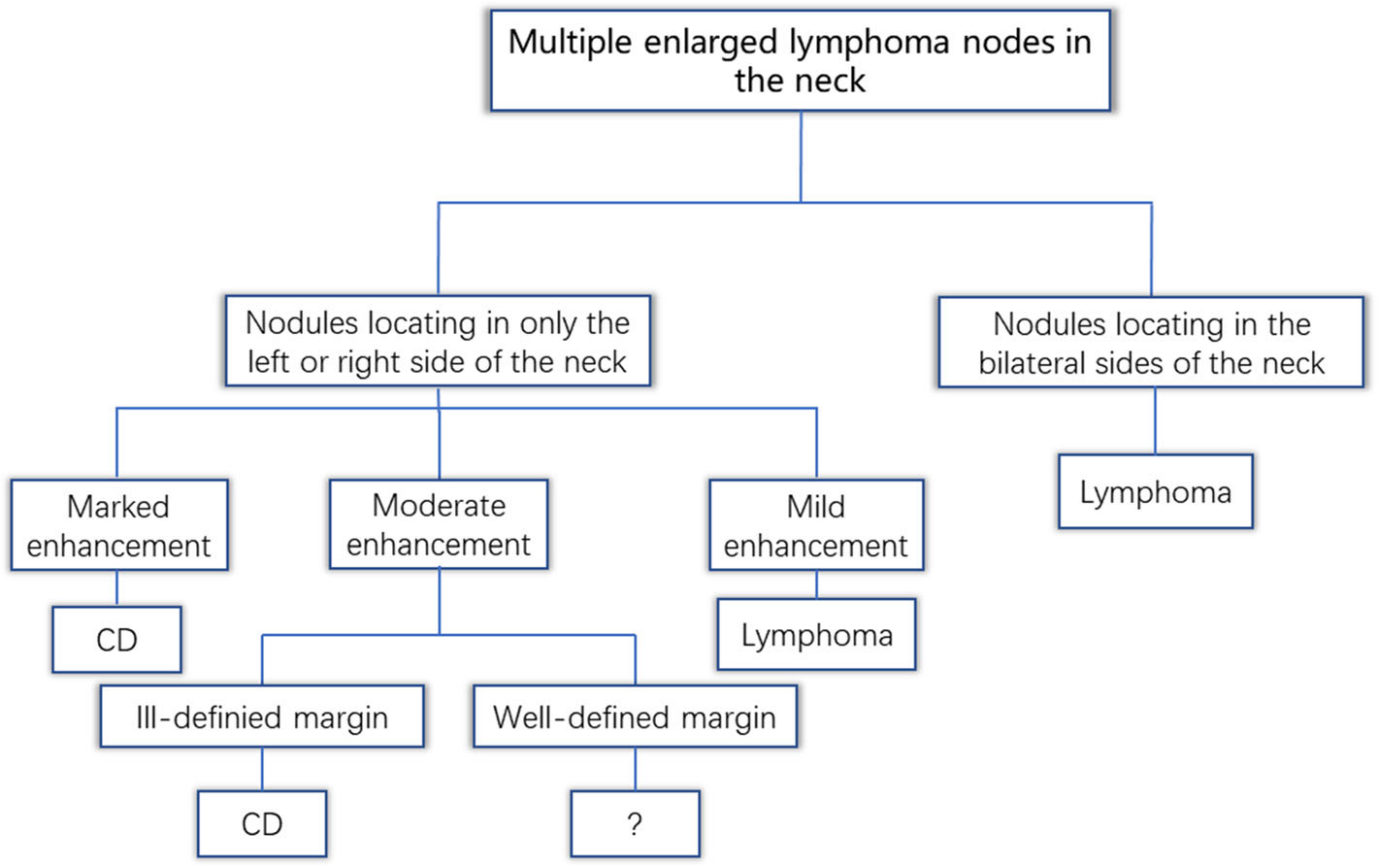
Decision tree analysis for differentiating CD and lymphoma with multiple lymph node involvement

**Fig. 9:**
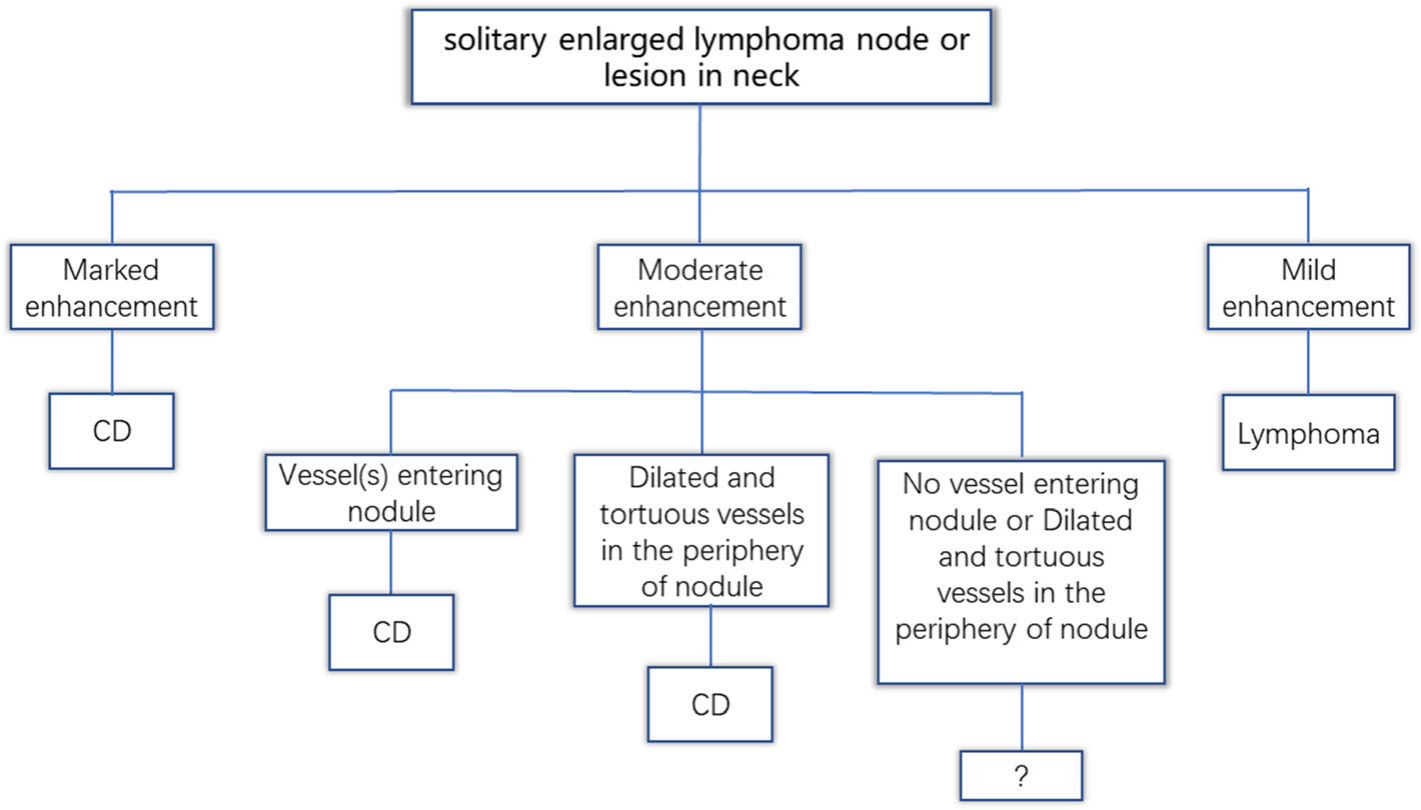
Decision tree analysis for differentiating CD and lymphoma with solitary lymph node involvement

The involvement of the neck lymph nodes showed several significant differences between the two conditions (Table 3). Notably, no marked enhancement was found in lymphoma patients, in contrast to marked enhancement being detected in 85.3% of CD cases (*p* < 0.001). Vessels entering a nodule were detected in 67.6% of CD cases (Fig. 3a–d), but none were detected in lymphoma cases. Dilated and tortuous vessels in the periphery of a nodule were significantly more frequent in CD than in lymphoma (*p* < 0.001; Fig. 1a–d). ROC analysis (Fig. 7) showed a significant difference in contrast-enhanced CT values between lymphoma and CD (*p* < 0.001, area under curve = 0.954), with an estimated cut-off value of 92.5 HU. HU values of nodules after enhancement were significantly higher in the CD patients than in the lymphoma patients. In addition, we constructed decision trees according to these imaging characteristics (Figs. 8 and 9).

Indicators of the diagnostic value of contrast-enhanced CT for CD are listed in Table 4.

**Table 4:** Diagnostic Value of CT Indicators for CD

Contrast-enhanced CT features	Sensitivity	Specificity	PPV	NPV	Youden Index
vessel(s) entering nodule (1)	75%	74%	55%	84%	0.49
Dilated and tortuous vessels in the periphery of nodule (2)	67%	79%	61%	92%	0.46
(1) + (2)	78%	93%	79%	91%	0.71
Degree of enhancement (3)	85%	78%	53%	93%	0.63
(1) + (2) + (3)	89%	94%	89%	94%	0.83

*Abbreviations: PPV* positive predictive value, *NPV* negative predictive value

## Discussion

CD is a rare neoplasm involving the lymph nodes, which may involve the mediastinum, abdomen, and neck, and accounts for approximately 8–12% of primary neck tumors. In the present study, we compared the contrast-enhanced CT characteristics of CD and lymphoma. At initial evaluation, CD usually presented as a solitary enlarged lymph node or nodule(s) with homogenous enhancement, while lymphoma typically manifested as multiple enlarged lymph nodes with moderate enhancement (78.2% of cases). Calcification and the presence of necrosis were rare in either condition.

We also detected several striking differences between CD and lymphoma, and in patients with CD or lymphoma manifesting as a solitary mass, the most significant difference was the degree of enhancement; while marked enhancement was not detected in lymphoma, most CD nodules (85.3%) showed marked enhancement. For CD and lymphoma patients presenting with multiple enlarged lymph nodes, the most striking difference was the location of the nodules. Bilateral involvement of the sides of the neck was not detected in any CD case. This single-sided location may be a striking characteristic of CD in patients with multiple nodules. Additionally, vessels entering nodules (67.6%) were frequently observed in CD, but were rare in lymphoma. Dilated and tortuous vessels in the periphery of a nodule were also more common in CD than in lymphoma.

Several reports have attempted to distinguish CD from benign nodules on CT imaging [4, 9, 11], with marked enhancement having been suggested as a meaningful CT feature for CD. On contrast-enhanced CT imaging, CD usually manifested as marked enhancement and occasionally as intermediate enhancement, with cysts and necrosis in the nodules being rare. Jiang et al. reported on 21 cases of CD [5], with 15 cases (71.4%) showing marked enhancement. Hill et al. reported on 26 patients with CD, with 21 (80.7%) of the patients demonstrating these typical CT imaging findings [14]. Our imaging findings of CD are in agreement with these previous reports, with the proportion of marked enhancement and intermediate enhancement in CD being 85.3% and 14.7% respectively in the present study. Only one CD case manifested as a non-homogeneous nodule with a central non-enhancing area of mild hypodensity. The imaging characteristics of this case were similar to one of the four Castleman disease cases reported by Park et al., who also reported a CD lesion with a central non-enhancing low-density area, which was attributed to a dense fibrous scar [15].

Lymphomas are primary neoplasms of the lymphoreticular system [16] and may be divided into two types according to their histopathologic classification: Hodgkin’s disease (HD) and non-Hodgkin’s lymphoma (NHL) [17]. HD often occurs in younger patients, while NHL is commonly seen in the Chinese population and occurs in a greater range of ages than HD [18]. In our series of lymphoma patients, most of the cases were NHL. In published articles, lymphoma generally presents as an isolated mass or enlarged lymph nodes, with homogeneous mild or moderate contrast-enhancement [19, 20]. Intra-nodular necrosis or cysts are only occasionally discovered in untreated lymphomas with a large diameter [21]. Our lymphoma subjects demonstrated similar imaging characteristics, with 43 (78.2%) cases presenting as moderate enhancement and 12 (21.8%) cases as mild enhancement. The presence of central necrosis was detected in only 2 (3.6%) cases with a large diameter, and was attributed to a lack of blood supply in the center of the lesions.

The presentation of patients with CD is variable, and depends on the subtype; CD can present along a spectrum ranging from an asymptomatic localized nodal mass, which is often discovered incidentally, to multifocal adenopathy with B-symptoms and hematological derangements that clinically mimic lymphoma. In our study, the patients with lymphoma presented significantly more frequently with systemic symptoms such as fatigue and fever [22], and the interval between the first contrast-enhanced CT and biopsy or surgery was significantly shorter in the lymphoma group than in the CD patients. The absence of systemic symptoms was more often detected in patients with the hyaline vascular (HV) subtype of CD, and CD is typically of the diffuse HV-CD type, only rarely presenting as other types such as plasma cell, HHV-8-associated, and multicentric type CD. Those may illustrate the phenomenon.

There are few reports on CT imaging of the vessels which surround or enter nodules of the two conditions. Although previous CT studies have focused on the degree of enhancement to distinguish CD from lymphoma [4, 16, 23, 24], the enhancement extent of the two entities sometimes overlaps. In our study, 5 (14.7%) cases with CD and 43 (78.2%) cases with lymphoma demonstrated moderate enhancement on initial contrast-enhanced CT. This study showed that dilated and tortuous vessels surrounding or entering nodules were significantly more often observed in CD than in lymphoma. The presence of these vessels can therefore be taken as suggesting the possibility of CD. In our study, 23 (67.6%) cases with CD showed arteries entering nodules and 12 (35.3%) cases demonstrated dilated and tortuous vessels surrounding nodules. The combination of dilated and tortuous vessels in the periphery of a nodule, and vessels entering a nodule, yielded high capability in the differential diagnosis, with sensitivity, specificity, and Youden index of 78%, 93%, and 0.71 respectively. In the lymphoma group, we also detected vessels surrounding the nodules in a small proportion of cases; however, most of the vessels were slender with a smaller diameter. Furthermore, no lymphoma case showed evidence of vessels entering nodules on CT imaging.

We found that an additional feature for helping to distinguish CD from lymphoma was the anatomical distribution of the nodules. In cases involving multiple lymph nodes, all the nodes were located in only the left or right side of the neck in CD cases, not bilaterally. Unilaterality of enlarged lymph nodes may help reach the correct diagnosis of CD on imaging.

Few articles have evaluated the CT attenuation values for differentiation of CD and lymphoma. In this study, ROC curves were used to compare the diagnostic performance of the attenuation values, with the area under the curve being 0.954; this value demonstrated good validity for the diagnosis of CD. The optimal cut-off point for differentiating CD from lymphoma was 92.5 HU, with values above this threshold being likely to represent CD.

The present study has several limitations. Because of its retrospective nature and the radiation dose to the patients, there was insufficient data available to evaluate advanced CT techniques such as CT perfusion. Second, the analysis of the degree of enhancement in neck nodules was partially performed by visual assessment. Two experienced head and neck radiologists reviewed all CT images to reduce bias in these visual assessments. We must also emphasize the general incidence of both conditions, with CD occurring less frequently than lymphoma. This makes the diagnosis of the two conditions more complex, as few contrast-enhanced CT features are able to make a clear differentiation between the conditions. We therefore attempted to combine relevant contrast-enhanced CT features to facilitate the differentiation and provide radiological clues for further work-up.

## Conclusions

Our findings suggest that at initial presentation, contrast-enhanced CT is suitable for differentiating between CD and lymphoma of the neck in many cases, with the differentiation being performed on the basis of enhancement patterns, nodule locations, attenuation values, and the presence of tortuous vessels in enlarged lymph nodes.

## Abbreviations

AIDS: Acquired immunodeficiency syndrome
CD: Castleman disease
HD: Hodgkin’s disease
HHV-8: Human herpes virus-8
HU: Hounsfield unit
HV-CD: Hyaline vascular CD
IL-6: Interleukin-6
NHL: Non-Hodgkin’s lymphoma
NPV: Negative predictive value
PPV: Positive predictive value
ROC: Receiver operating characteristic
ROI: Region of interest
